# Supplementary material for: SParticle, an algorithm for the analysis of filamentous microorganisms in submerged cultures
Source: Antonie Van Leeuwenhoek. 2017 Sep 15;111(2):171–82. doi: 10.1007/s10482-017-0939-y (PMC5772119; doi:10.1007/s10482-017-0939-y)
Supplement: Supplementary file 1 — Supplementary material 1 (PDF 382 kb) [file 10482_2017_939_MOESM1_ESM.pdf]

SUPPLEMENTAL INFORMATION for

SParticle, an algorithm for the analysis of filamentous microorganisms in submerged cultures

Joost Willemse[1], Ferhat Büke[1], Dino van Dissel[1], Sanne Grevink[1], Dennis Claessen[1], Gilles P. van Wezel[1,2]

[1]Molecular Biotechnology, Institute of Biology, Leiden university, Sylviuswewg 72, Leiden 2333BE, The Netherlands.

[2]Corresponding author: g.wezel@biology.leidenuniv.nl

Contents:

Supplemental Methods

Supplemental FIgures

References

SUPPLEMENTAL METHODS

Voronoi explanation

The borders of these regions are determined based on the ROIs specified beforehand, and for each ROI there is a corresponding region consisting of all points closer to that ROI than to any other ROI. The determined region borders are used as to identify separation lines. In the end this transformation was subtracted from the original thresholded binary image to separate touching objects.

Filter settings for identifying mycelial pellets

Morphological number above 0.85. The morphological number is calculated and is used to describe the shape of objects and is a combination of Area, Solidity, Feret diameter, and aspect ratio (AR) (Wucherpfennig, Hestler et al. 2011).

Area greater than 10.000 pix & AR greater than 3. This eliminates big and very non-circular areas. These are generally cotton fibers.

Circularity greater than 0.4 & Area less than 10.000 pix. Small circular areas, generally small air bubbles and out of focus pellet fragments.

Area less than 50.000 pix & SDB greater than 50. Areas that are as big as a small-medium pellet with high SDB. These are medium sized areas that are badly focused, heterogeneously bright areas.

Area greater than 1.000 pix & SDB less than 15. This corresponds to areas bigger than mycelia fragments that are blurry.

Area less than 1.000 pix & Round greater than 0.7. These are small circular areas. Areas that small are mycelia fragments and should not be circular since they are supposed to be filamentous.

Area greater than 10.000 % SDB less than 20. Big areas that are out of focus and therefore blurry.

Mean greater than 200. Highly bright areas. Pellets or mycelia, since being heterogeneous, should not reach these high levels of brightness on average.

SDB greater than 55. Areas which are highly heterogeneous in brightness. No mycelia or pellet has that high a difference in between bright and dark pixels.

SDB less than 12. Very homogeneous areas. These are out of focus areas. Pellets or mycelia fragments are generally more heterogeneous because of phase contrast imaging.

Mean greater than 110 and SDB less than 25 OR Mean greater than 75 and SDB less than 22. These rules check for heterogeneity of brightness in images given that their brightness is greater than 75 or 110. As brightness increase the tolerance for heterogeneity increases.

Mean greater than 75 and Round less than 0.3. Average and above brightness areas that are not circular cannot be pellets. Because in phase contrast small areas are darker and big areas are on average brighter. Since big areas pellets and they have a close shape they should be more circular. Therefore bright areas that are not round are eliminated.

Mean greater than 100 and Circularity less than 0.09. Again checks for circularity on bright areas using another measurement of circularity other than roundness.

Round greater than 0.7 and SDB less than 25. Round and homogeneous areas. These are generally out of focus bubbles which cannot directly be identified via circularity and AR.

Area less than 10.000pix and Round greater than 0.8 and Mean greater than 100. Small areas which are highly round. These are generally bubbles that could not have been identified via AR or Circularity.

Area greater less than 2.500pix and Circularity greater than 0.1 and Mean greater than 100. This is areas that are small and that have both high circularity and high roundness values. This captures any bubble that is left from other rules as well as out of focus small circular areas.

Focus check. After previous rules are checked for all ROI's, remaining areas are looked at one more time using focus controlling. This is done by identifying the edges of the ROI and applying an "Edge Finding" transformation. A band on the edge of the area is taken and measured for average intensity. If the edge is well defined in the image, the Edge Finding

transformation should return a high value. By applying a threshold, any unfocused region that has been missed with previous filters was found and eliminated from measurements.

Data export

Data gathered in ImageJ were stored in the temporary result table but also in the folder from which the image that is analyzed originates. Therefore, the identified objects' measurements were sorted from small to large according to the area and written into a csv file. This csv file can be automatically opened in Microsoft Excel® along with a macro file which automatically draws important parameter graphs. These are interactive graphs linked to a small image file with the size of the bounding box of the ROI from the original image, which is created automatically during measurements. This represents a useful way to review the measured images, allowing a quick way to pull images representing a subpopulation or assessing the morphology of outliners and can also be used to select images for publications. Additionally, all discarded images are stored with specific details as to why they were discarded in the image name, allowing refinement of the filtering parameters.

Explanation of polar circularity

Pellets often have hyphae protruding outwards, and thus have a large perimeter length as compared to the surface area. Therefore, methods that calculate the circularity using the perimeter length would not give an accurate description of the shape. After Cartesian to Polar coordinate transformation, a perfect circle becomes a line in Radius vs. Angle graph, while any shape deviating from a circle in Cartesian coordinates deviates from a line in polar coordinates (Stojmenovic *et al.* 2013). For example, in a perfect circle the distance between the center and the border is equal for any angle. While in a square of 2 by 2 for example the distance from the center in relation to the angle will change from 1 (0 degrees) to the square root of 2 (45 degrees), and recede to 1 again for a 90 degree angle, and so on (More information in Fig. S2). Based on the standard deviation from the average radius we can distinguish different shapes. The standard deviations from the line calculated and divided by

the average distance value to correct for shape’s size resulting in the polar circularity value. The larger the polar circularity value the more the actual object radius deviates from the average radius, in other words, a shape that is less circular. While a perfect circle would have a value of 0, the value increases as the shape deviates from a circle.

SUPPLEMENTAL FIGURES

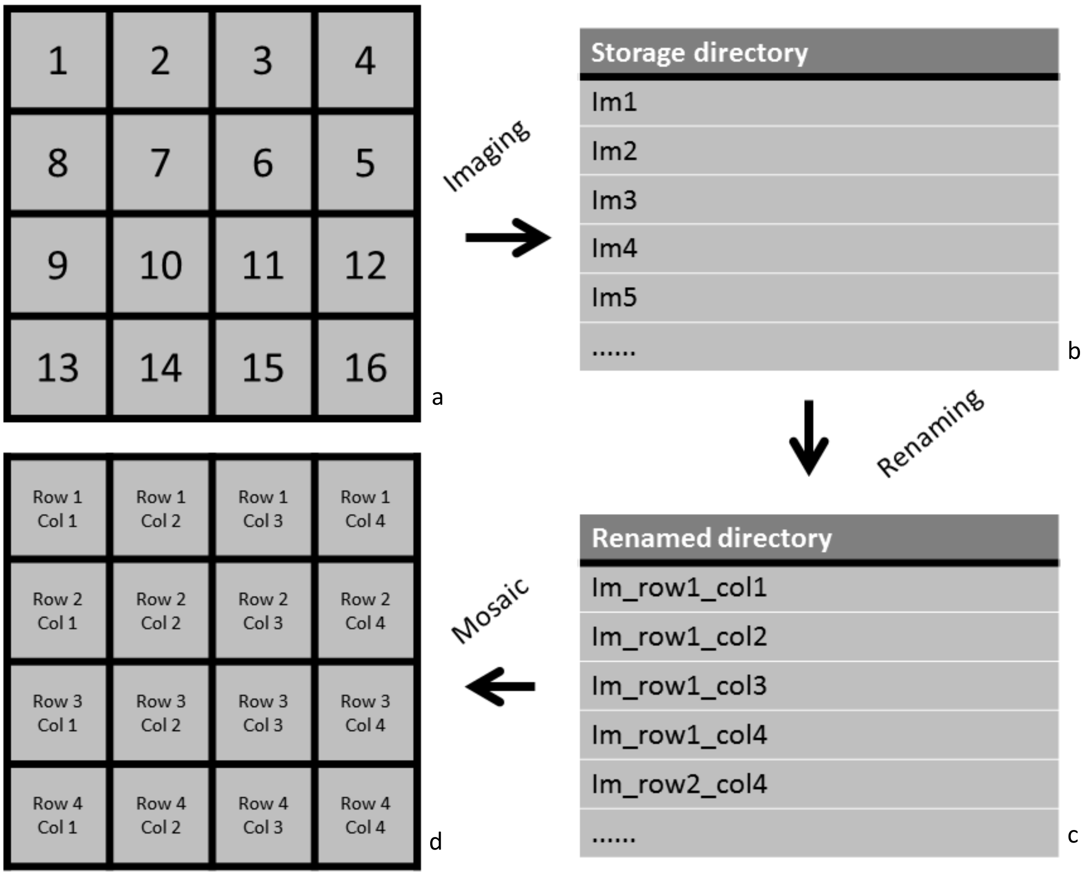

Fig. S1 Workflow from imaging to reconstructed mosaics in ImageJ. The workflow starts with automated image capturing. (a) The odd rows are captured from left to right, the even rows from right to left, meandering over the image. Numbers in the cells indicate the capture sequence. (b) All images are stored separately as tiff files. (c) Based on the image number and the given mosaic size (user input), the files are renamed and the mosaics are reconstructed. (d) The size of the mosaic is determined by memory availability.

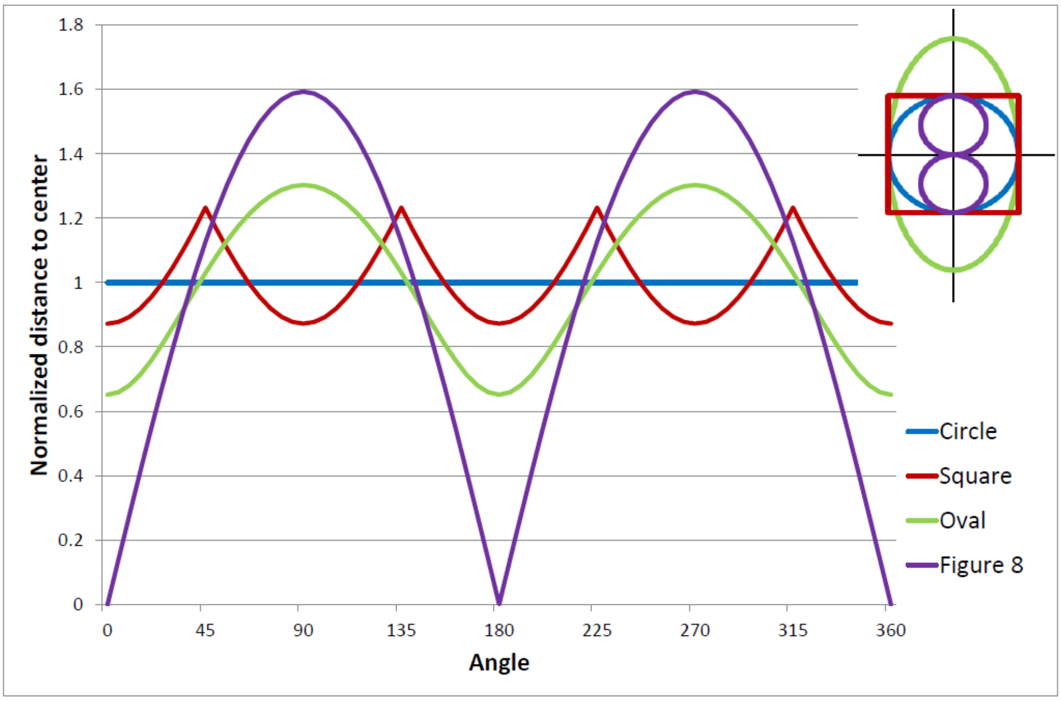

Fig. S2. Center to border distances of different shapes showing that Cartesian to polar coordinates can reveal differences in circularity between objects. The figure shows that perfect circles do not deviate from the radius at any point (standard deviation from r = 0). Squares of the same diameter have an increased deviation (standard deviation from r = 0.11), an oval with double the length of the width gives a larger deviation (standard deviation from r = 0.23). The figure of 8 deviates even more (standard deviation from r = 0.51). This demonstrates that shape deviations from a perfect circle can be identified using polar coordinates.

References

Stojmenovic M, Jevremovic A, Nayak A Fast iris detection via shape based circularity. In: Industrial Electronics and Applications (ICIEA), 2013 8th IEEE Conference 2013. pp 747-752.
